# Research advances in huntingtin-associated protein 1 and its application prospects in diseases

**DOI:** 10.3389/fnins.2024.1402996

**Published:** 2024-06-21

**Authors:** Yongjiang Wu, Yanfei Wang, Yunchi Lu, Junguo Yan, Hongjun Zhao, Riyun Yang, Jingying Pan

**Affiliations:** Department of Histology and Embryology, Medical School of Nantong University, Nantong, China

**Keywords:** huntingtin-associated protein 1, neurological diseases, cancer, diabetes mellitus, neuropathology

## Abstract

Huntingtin-associated protein 1 (HAP1) was the first protein discovered to interact with huntingtin. Besides brain, HAP1 is also expressed in the spinal cord, dorsal root ganglion, endocrine, and digestive systems. HAP1 has diverse functions involving in vesicular transport, receptor recycling, gene transcription, and signal transduction. HAP1 is strongly linked to several neurological diseases, including Huntington’s disease, Alzheimer’s disease, epilepsy, ischemic stroke, and depression. In addition, HAP1 has been proved to participate in cancers and diabetes mellitus. This article provides an overview of HAP1 regarding the tissue distribution, cell localization, functions, and offers fresh perspectives to investigate its role in diseases.

## 1 Introduction

Li et al. (1995) discovered a previously unidentified protein by yeast double hybridization that interacts with huntingtin (Htt), which was subsequently named huntingtin-associated protein 1 (HAP1) (Li et al., 1995). The interaction between HAP1 and Htt depends on the length of polyglutamine (polyQ). HAP1 has a stronger binding affinity to mutant huntingtin (mHtt) than Htt (Li et al., 1995; Metzger et al., 2008). In neurons, HAP1 is mainly distributed in the cytoplasm, axons, dendrites, microtubules, and microfilaments (Li et al., 2000; Xiang et al., 2014). HAP1 is also distributed in membranous organelles, such as mitochondria, the endoplasmic reticulum, lysosomes, synaptic vesicles, and tubular vesicles. It is worth noting that HAP1 is seldom expressed in cell nuclei (Gutekunst et al., 1998; Marcora and Kennedy, 2010). Two isoforms of HAP1, HAP1A (579–599 aa, ∼75 kD) and HAP1B (579–629 aa, ∼85 kD), have been identified in the rat brain (Li et al., 1995). Only one HAP1 isoform has been identified in the primate brain, with a relative molecular mass of approximately 75 kD; this is similar to the size of HAP1A in rodents (Li et al., 1998c), which is expressed mainly in the hippocampus, amygdala, and caudate nucleus (Li et al., 1996; Chen et al., 2022). By light microscopy, HAP1-immunoreactive products appear as a stigmoid body (STB), a specialized cellular structure with a diameter of 0.5–3 μm (Gutekunst et al., 1998; Li et al., 1998a). STBs are formed by multiple fusions of intracellular microtubules (Fujinaga et al., 2009). *In vitro*, transfection of HAP1A results in the formation of STBs, while HAP1B is expressed diffusely in the cytoplasm. The C-terminus of HAP1A and HAP1B promotes and inhibits STBs formation, respectively (Fujinaga et al., 2007). HAP1/STBs has been suggested to protect against apoptosis triggered by aberrantly amplified mHtt and polyQ (Fujinaga et al., 2011).

In physiology, HAP1 is crucial for regulating gene transcription, vesicular transport, membrane endocytosis, receptor recycling, calcium ion activity, and other functions (Wu and Zhou, 2009; Zhao et al., 2022). In pathology, HAP1 is associated with a variety of diseases, such as neurological diseases, cancers, and diabetes mellitus. This review details HAP1 interacting proteins, its distribution and role in different tissues. This detailed exploration of the possible correlation between HAP1 and diseases aims to provide new insights and objectives for diagnosis and therapy.

## 2 Distribution of HAP1

**FIGURE 1:**
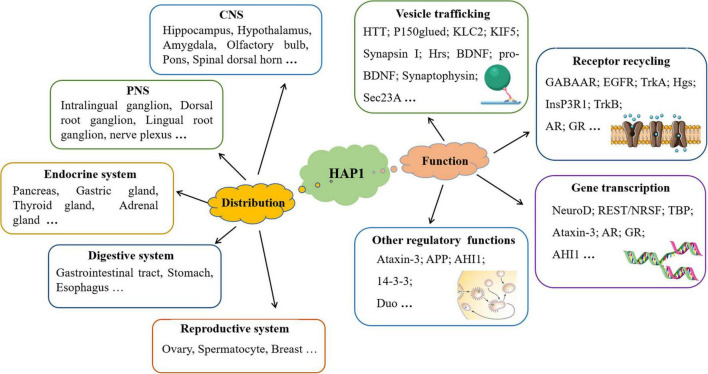
The distribution and function of HAP1 are discussed in this manuscript. In addition to being expressed in the nervous system, HAP1 is also expressed in the endocrine, digestive, and reproductive systems. HAP1 greatly participates in multiple processes through interactions with various proteins.

Although initial research on HAP1 demonstrated its expression only in the central nervous system (Li et al., 1995), recent studies have shown that HAP1 is present in various organ systems, including the endocrine, digestive, and reproductive systems, where it participates in processes such as insulin secretion (Figure 1).

### 2.1 Central nervous system

Huntingtin-associated protein 1 is highly expressed in certain regions of the brain, including olfactory bulb, hippocampus, amygdala, caudate nucleus, and some brainstem nuclei, particularly in the hypothalamus supraoptic nucleus, paraventricular nucleus, and arcuate nucleus (Fujinaga et al., 2004; Chen et al., 2020). In rodents, HAP1 mRNA expression in the hypothalamus is nearly 10 times greater than that in other brain regions, while in monkeys, it is approximately 2–3 times greater (Chen et al., 2022). HAP1 is predominantly expressed in the gray matter surrounding the posterior horn and central canal of the spinal cord (Liao et al., 2005), particularly in superficial layers I and II of the posterior horn, where HAP1 immunoreactivity is strongest, which indicates its potential role in pain regulation (Dragatsis et al., 2000; Islam et al., 2017). The detailed distribution of HAP1 in the CNS is shown in Table 1.

**TABLE 1:** The expression and distribution of HAP1 in the central nervous system (CNS).

Location	Tissues or cells	Expression level	Resources
Telencephalon	Olfactory bulb	High	Rat; mouse (Li et al., 1996; Fujinaga et al., 2004)
	Cerebral cortex	High to moderate	Human; monkey; rat; mouse (Li et al., 1996; Page et al., 1998)
	Hippocampus	High to moderate	Human; monkey; rat; mouse (Li et al., 1996; Page et al., 1998; Fujinaga et al., 2004)
	Septal area	Moderate	Mouse (Page et al., 1998; Fujinaga et al., 2004)
	Amygdala	High	Human; rat; mouse (Li et al., 1996; Page et al., 1998; Fujinaga et al., 2004)
	Corpus striatum	High to moderate	Human; monkey (Li et al., 1996)
	Corpus striatum	Low	Rat; mouse (Page et al., 1998; Fujinaga et al., 2004)
	Basal forebrain	Low	Mouse (Fujinaga et al., 2004)
White matter	Extremely low	Human; monkey; rat (Li et al., 1996; Fujinaga et al., 2004)
Diencephalon	Hypothalamus	High	Rat; mouse (Page et al., 1998; Fujinaga et al., 2004)
	Thalamus	Extremely low	Human; rat (Li et al., 1996; Fujinaga et al., 2004)
Cerebellum	Cerebellum	Moderate	Human; rat; mouse (Li et al., 1996; Fujinaga et al., 2004)
Brain stem	Medulla oblongata	High to moderate	Rat; mouse (Fujinaga et al., 2004; Islam et al., 2022)
	Pons	High to moderate	Mouse (Fujinaga et al., 2004; Islam et al., 2022)
	Midbrain	High to moderate	Rat; mouse (Li et al., 1996; Fujinaga et al., 2004; Islam et al., 2022)
Spinal cord	Dorsal horn	High to moderate	Rat; mouse (Page et al., 1998; Dragatsis et al., 2000; Islam et al., 2017; Pan et al., 2023)

Notably, HAP1 expression patterns differ significantly between rodent and primate brains. In rodents, HAP1 is defective in brain regions such as the striatum, hippocampus, and neocortex, where neuronal loss is most severe in patients with Huntington’s disease (HD) (Fujinaga et al., 2004). These regional differences in HAP1 expression suggest that HAP1 may have a protective function against neurodegeneration (Fujinaga et al., 2004; Wroblewski et al., 2018). In contrast, HAP1 is widely distributed throughout various regions of the primate brain, mirroring the distribution of Htt (Chen et al., 2022). Besides these distribution differences, HAP1 functions differently in the brains of these two species. In mice, loss of HAP1 impacts brain development and postnatal survival, but it does not affect neuronal differentiation or gene expression in developing human neurons (Chan et al., 2002; Li et al., 2003; Chen et al., 2022). In primates, HAP1 deficiency seems to be compensated by Htt. However, in both mice and monkeys, HAP1 deficiency worsens the neurotoxicity of mHtt, leading to striatal neuronal death (Liu et al., 2020; Chen et al., 2022). Further research is needed to explore the differences in HAP1 expression and function as well as its role in neuronal dysfunction in the primate brain.

### 2.2 Peripheral nervous system

Huntingtin-associated protein 1 is distributed within small and medium-sized neurons in dorsal root ganglia (DRG) (Islam et al., 2020; Pan et al., 2023). Double immunostaining for HAP1 and markers of nociceptive or mechanoreceptive neurons revealed that approximately 70%–80% of CGRP-, SP-, CB-, NOS-, TRPV1-, CR- and PV-immunoreactive neurons express HAP1 (Islam et al., 2020). HAP1 is highly expressed in nociceptive/ proprioceptive neurons, which suggests that it plays an important role in pain transduction and proprioception. HAP1 is also expressed in the rodent enteric nervous system, and though its expression does not differ between different intestinal segments, it is more highly expressed in rats than in mice (Tarif et al., 2023). In the submucosal nerve plexus, HAP1 is expressed in secretomotor and vasodilator neurons (Tarif et al., 2023), whereas in the myenteric nerve plexus, HAP1 is predominantly expressed in motor neurons (Tarif et al., 2021). The detailed distribution of HAP1 in the peripheral nervous system (PNS) is shown in Table 2.

**TABLE 2:** Expression and distribution of HAP1 in the peripheral nervous system (PNS).

Location	Tissues or cells	Expression level	Resources
Dorsal root ganglion	Neurons	Moderate	Rat; mouse (Islam et al., 2020; Pan et al., 2023)
Intralingual ganglion	Neurons	Abundant distribution	Mouse (Islam et al., 2023)
Lingual root ganglion	Neurons	Abundant distribution	Mouse (Islam et al., 2023)
Submucosal nerve plexus	Neurons	High	Rat; mouse (Tarif et al., 2023)
Myenteric nerve plexus	Neurons	High	Rat; mouse (Tarif et al., 2021)
Abdominal sympathetic ganglia	Neurons	Extremely low	Mouse (Dragatsis et al., 2000)
Intestinal neurons	Neurons	Extremely low	Mouse (Dragatsis et al., 2000)

### 2.3 Endocrine system

Within the endocrine system, HAP1 is distributed throughout the anterior and posterior pituitary, thyroid, and adrenal medulla (Liao et al., 2005; Mackenzie et al., 2014). HAP1 is present in nitrogen-containing hormone-secreting cells but absent in steroid hormone-secreting cells, which indicates a strong correlation between HAP1 and nitrogen-containing hormone production (Liao et al., 2005; Fujinaga et al., 2011). HAP1 is selectively expressed in pancreatic islet β-cells, and approximately 80% of β-cells express both HAP1 and insulin (Liao et al., 2010; Pan et al., 2016), which suggests a potentially important role for HAP1 in hormone secretion by endocrine cells.

The gastrointestinal tract is an important part of the endocrine system. A study of rat pyloric glands revealed that HAP1 is specifically expressed in gastrin (G) cells (Yanai et al., 2020), which are neuroendocrine cells responsible for gastrin synthesis and secretion (Schubert, 2016), suggesting the possible involvement of HAP1 in the regulation of gastrin secretion. Enterochromaffin (EC) cells, which are the most abundant of all enteroendocrine cells, synthesize and secrete 95% of the 5-hydroxytryptamine (5-HT) (Rezzani et al., 2022). In EC cells, HAP1 is expressed and colocalizes with 5-HT (Lumsden et al., 2016), indicating that HAP1 is involved in 5-HT secretion. The detailed distribution of HAP1 in the endocrine system is shown in Table 3.

**TABLE 3:** The expression and distribution of HAP1 in the endocrine system.

Location	Tissues or cells	Expression level	Resources
Pituitary gland	Anterior lobe	High	Rat (Liao et al., 2005)
	Posterior lobe	Extremely low	Rat (Liao et al., 2005)
	Pars intermedia	High	Rat (Liao et al., 2005)
Thyroid gland	Solitary cells or small clusters	Moderate	Rat (Liao et al., 2005)
Adrenal glands	Adrenal chromaffin cells	Moderate	Rat (Liao et al., 2005; Mackenzie et al., 2014)
Pancreatic islets	B cells	High	Rat (Liao et al., 2005, 2010; Cape et al., 2012)
Stomach glands	G cells	High	Rat (Yanai et al., 2020)
Duodenum	Chromaffin cells	Moderate	Human (Lumsden et al., 2016)

### 2.4 Digestive system

Huntingtin-associated protein 1 is expressed in the digestive tract of both humans and rats (Zhao et al., 2022). Immunohistochemical analysis has shown that HAP1 is present in the mucosa throughout the gastrointestinal tract, with varying levels of expression in different regions (Li et al., 2019). The stomach has the highest expression, followed by the esophagus, while the small intestine has the lowest expression (Li et al., 2019). These differences may be linked to the distinct digestive functions of each region, as the stomach is crucial for the secretion of gastric acid, enzymes, and hormones. HAP1-positive cells are predominantly located in the mucosal gastric glands of the rat stomach, particularly at the base of the glands (Yanai et al., 2020). Regional differences in the number of HAP1-positive cells have also been reported: minimal numbers in the cardia glands, moderate numbers in the fundic glands, and abundant HAP1-positive cells in the pyloric glands (Liao et al., 2005). In the rat small intestine, the number of HAP1-positive cells is lower in the intestinal villi and intestinal glands than in the stomach (Liao et al., 2005).

### 2.5 Reproductive system and retinal tissues

Huntingtin-associated protein 1 is also expressed in the reproductive system and in retinal tissues (Liao et al., 2005). HAP1 is found in mouse testicular spermatocytes, ovaries, and human mammary glands (Dragatsis et al., 2000; Zhu et al., 2013). Notably, HAP1 expression in breast cancer tissues is one-third lower than that in normal breast tissues (Zhu et al., 2013). This indicates that HAP1 is a potential biomarker in breast cancer. In addition, HAP1 is highly expressed in the ganglion cell layer, internal and external nuclear layers of the retina (Liao et al., 2005).

## 3 Functions of HAP1

Huntingtin-associated protein 1 can participate in multiple physiological and pathological processes through interacting with many proteins, such as vesicle transport and secretion, membrane endocytosis, receptor recycling, signal transduction, and gene transcription (Figure 1).

Substantial evidence indicates that HAP1 is crucial for vesicular transport in neurons and axons, where it plays a key role in both anterograde and retrograde transport. HAP1 interacts with the dynamin p150Glued subunit, and the binding of these two proteins induces retrograde transport in microtubule-dependent membrane organelles (Engelender et al., 1997; Li et al., 1998b). Similarly, HAP1 also interacts with kinesin light chain (KLC), and reducing HAP1 expression inhibits kinesin-dependent translocation of amyloid precursor protein (APP)-containing vesicles (McGuire et al., 2006). In addition, HAP1 acts as an adapter that connects the γ-aminobutyric acid type A receptor (GABA_A_R) to kinesin family motor protein 5 (KIF5) and controls GABA_A_R transport along microtubules in dendrites (Yuen et al., 2012; Twelvetrees et al., 2019). These findings suggest that HAP1 is a scaffold protein that mediates the interaction between vesicles and motor proteins involved in intracellular transport (Twelvetrees et al., 2019). HAP1 interacts with these microtubule-dependent transporter proteins to promote the localization of HAP1 to the tip of the neurite. In contrast, phosphorylation of HAP1 reduces its binding to p150Glued and KLC and decreases its localization at the neurite tip (Rong et al., 2006). In one study, inhibition of HAP1 expression by RNA interference reduced neural protrusion growth in PC12 cells (McGuire et al., 2006). Other studies revealed that 14-3-3 reduced the binding of HAP1A to KLC, decreased the transport of HAP1-A to neuronal protrusions and inhibited the role of HAP1A in promoting neurite growth (Rong et al., 2007; Wen et al., 2022). Furthermore, it has been shown that the Htt/HAP1/p150Glued complex is involved in brain-derived neurotrophic factor (BDNF) vesicular transport (Gauthier et al., 2004). HAP1 also interacts with the precursor of BDNF (pro-BDNF) and modulates its trafficking, processing and degradation (Wu et al., 2010; Yang et al., 2011). In addition, HAP1 interacts with proteins such as abelson helper integration site-1 (AHI1), breakpoint cluster region protein, synaptophysin, pericentromeric protein 1, Duo, clathrin light chain B, and sec23A, and is involved in intracellular transport, endocytosis, and cytokinesis, which regulate the cytoskeleton, synaptic signaling and free radical production (Colomer et al., 1997; Engelender et al., 1997; Sheng et al., 2008; Huang et al., 2015; Mackenzie et al., 2016, 2017).

Huntingtin-associated protein 1 is also involved in the regulation of membrane receptor recycling and participates in signal transduction. HAP1 has been shown to interact with various proteins, such as the GABA_A_R subunit, AHI1, and hepatocyte growth factor-regulated tyrosine kinase substrate (Hgs), and plays a role in preventing the degradation of receptors such as GABA_A_R, tyrosine kinase receptor B (TrkB), epithelial growth factor receptor (EGFR), and tyrosine kinase receptor A (TrkA) (Klein et al., 1993; Li et al., 2002, 2003; Kittler et al., 2004; Rong et al., 2006). These interactions with HAP1 enhance the stability of internalized receptors and facilitates their recycling and return to the plasma membrane. Similarly, HAP1 interacts with GR and stabilizes GR in the cytoplasm (Chen et al., 2020). HAP1 can also interact with inositol (1,4,5)-triphosphate receptor 1 (InsP3R1), affecting the activities of receptors and neurons (Márquez-Moñino et al., 2024). Htt and HAP1 bind to the C-terminus of InsP3R1 to form a ternary complex (Tang et al., 2003). HAP1A regulates neuronal cell death and energy metabolism by facilitating the interaction between Htt and InsP3R1, thus enhancing the responsiveness of InsP3R1 to InsP3 (Tang et al., 2004). HAP1 also interacts with transcription factors to regulate transcription, such as TATA sequence-binding protein (TBP) and neurogenic differentiation (NeuroD) (Prigge and Schmidt, 2007). HAP1 is crucial for regulating the activation of NeuroD via binding with Htt and MLK2. Mixed-lineage kinase 2 (MLK2) is a protein kinase that activates the JNK signaling pathway through phosphorylation of MKK4/7 (Marcora et al., 2003; Jiang et al., 2024).

In addition, HAP1 forms a complex with the transcriptional repressor RE1-silencing transcription factor/neuron-restrictive silencer factor (REST/NRSF), p150Glued, and Htt, which are involved in the REST/NRSF nuclear translocation process (Shimojo, 2008).

## 4 Advances in HAP1 and diseases

**FIGURE 2:**
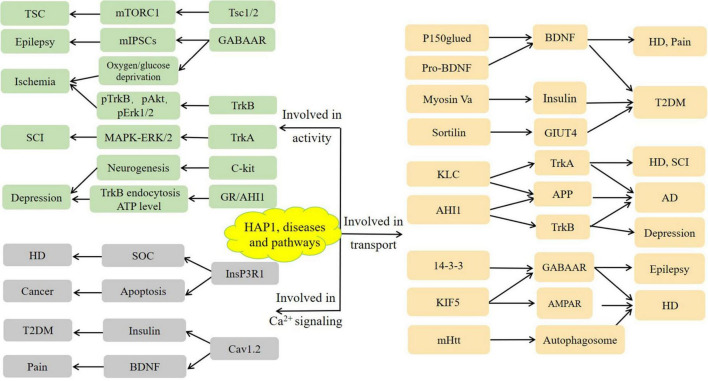
Illustration of diseases and pathways related to HAP1 discussed in this manuscript.

An increasing number of studies have revealed that the expression and function of HAP1 are associated with various diseases, including nervous system disorders, cancer, and diabetes (Figure 2).

### 4.1 Neurodegenerative disease

#### 4.1.1 Huntington’s disease

Huntington’s disease is an inherited, autosomal dominant monogenic neurodegenerative disorder characterized by choreiform involuntary movements with progressive cognitive and psychiatric deficits (Grosso Jasutkar and Yamamoto, 2021; Jia et al., 2022). Amplification of an abnormal CAG trinucleotide repeat sequence on IT15 of the Htt gene on chromosome 4 leads to the production of mHtt in affected individuals (Li et al., 1995). This protein accumulates in the brain and induces neurotoxicity, which causes severe loss of neurons in the striatum, hippocampus, and cerebral neocortex (Arteaga-Bracho et al., 2016; Mehler et al., 2019).

Huntingtin-associated protein 1 interacts with Htt and transporter proteins, and participates in intracellular transport. Due to its stronger binding affinity to mHtt than to normal Htt, the abnormal interactions of HAP1 with mHtt interfere with HAP1-dependent transport in various vesicles or via certain receptors, which results in the onset of neurodegenerative diseases. For example, mHtt can cause dissociation of the Htt/HAP1/p150Glued complex from microtubules, while the interaction of HAP1 with pro-BDNF is attenuated, which affects BDNF transport and release (Gauthier et al., 2004). Immunoprecipitation of pro-BDNF and HAP1 is reduced in brain homogenates from mouse models of HD (Wu et al., 2010). HAP1 is essential for BDNF endocytosis and signaling and for BDNF receptors in neurons (Lim et al., 2018). Dysregulation of BDNF, an essential pathological characteristic of HD, leads to a loss of neurotrophic support and increased neuronal toxicity (Simmons, 2017; Speidell et al., 2023). mHtt decreases the association of HAP1 with p150Glued and KLC, thereby decreasing the intracellular levels of TrkA (Rong et al., 2006), which is required for the internalization and transport of TrkA for neuronal protrusion growth. The trafficking of GABA_A_R and AMPA receptors, which are responsible for inhibitory and excitatory postsynaptic currents, respectively, is likewise hindered in an HD mouse model (Mandal et al., 2011; Yuen et al., 2012). HAP1 interacts with KIF5 to facilitate the trafficking of GABA_A_Rs and AMPARs along dendritic microtubules. This transport is hindered by mHtt, which causes an imbalance between excitatory and inhibitory signals in HD (Twelvetrees et al., 2010). Increased expression of mHtt leads to the aberrant interaction of HAP1 with Hgs, which results in aberrant endocytosis-based transport (Li et al., 2002). In addition, HAP1 acts as a dynamin-activated junction that drives autophagosome transport. mHtt interferes with the control of autophagosome movement by HAP1, resulting in impaired breakdown of cargo and buildup of mHtt in the neurons of individuals with HD (Wong and Holzbaur, 2014; Cason et al., 2021). This evidence suggests that mHtt impacts several HAP1-associated intracellular transport processes and is crucial for neurodegeneration.

Alterations in intracellular Ca^2+^ signaling pathways caused by HAP1 are believed to contribute substantially to the pathogenesis of HD (Kolobkova et al., 2017). In medium spiny neurons (MSNs), an InsP3R1-HAP1A-Htt ternary complex is formed that collectively participates in the regulation of Ca^2+^ signaling (Tang et al., 2003). mHtt sensitizes MSNs to InsP3R1 and promotes InsP3R1-mediated intracellular Ca^2+^ release (Czeredys et al., 2018). HAP1 is a necessary intermediate molecule for this process and can also promote the release of Ca^2+^ through the above process (Tang et al., 2004). In addition, store-operated calcium entry (SOCE) is a cellular process that is dysregulated in HD (Prakriya and Lewis, 2015). In the MSNs of YAC128 mice, HAP1A activates SOC channels by modulating IP3R1 activity (Czeredys et al., 2018).

The hallmark neuropathologic alteration in HD is severe degeneration of the striatum (Vonsattel et al., 1985), and HAP1 and other proteins that interact with Htt contribute to striatal neurodegeneration. Rhes, a GTPase that is abundantly expressed in the striatum and binds selectively to mHtt, is a candidate protein that promotes selective neuronal loss in the striatum of HD patients (Subramaniam and Snyder, 2011). Rhes enhances the sumoylation of mHtt and causes an increase in soluble mHtt, which leads to striatal neuronal death (Lee et al., 2014). The binding of HAP1 to mHtt prevents the binding of Rhes to mHtt and the sumoylation of mHtt, thereby attenuating the toxicity of mHtt (Liu et al., 2020). Patients with HD may benefit from an increase in HAP1 expression in striatal neurons, as this might result in the alleviation of symptoms.

#### 4.1.2 Alzheimer’s disease

Alzheimer’s disease (AD) is a progressive neurological condition with a gradual onset that often develops in older individuals, and its typical symptoms include progressive cognitive impairment and behavioral abnormalities (Soria Lopez et al., 2019). Amyloid precursor protein (APP) promotes the growth of neuronal synapses and is crucial for neuronal maturation, plasticity, and regeneration, but its cleavage results in the production of amyloid β protein (Aβ), which forms extracellular plaques. The development of synaptic damage and neurodegeneration results from the buildup of extracellular Aβ plaques and intracellular neurofibrillary tangles (Bloom, 2014).

Accumulating evidence indicates that neurodegeneration is a result of mutations in proteins involved in microtubule-dependent intracellular transport (Anderson et al., 2014; Vicario-Orri et al., 2015). Decreased microtubule-dependent transport can result in increased levels of Aβ and the accumulation of amyloid plaques in AD. Kinesin-dependent APP axonal transport in neurons is influenced by HAP1, and inhibition of HAP1 expression or deletion of the HAP1 gene suppresses kinesin-dependent transport of vesicles containing APP (McGuire et al., 2006). In addition, HAP1 and APP are highly colocalized and interact in many brain regions. HAP1 promotes the activation of nonamyloidogenic pathways and negatively regulates Aβ production in neurons (Yang et al., 2012). Knockdown of HAP1 significantly alters vesicular transport and endocytosis of APP, which decreases the reinsertion of APP into the cytoplasmic membrane and ultimately increases the level of Aβ and promotes disease progression (Yang et al., 2012). AHI1, a protein that interacts with HAP1, is also involved in APP transport and processing and reverses the typical pathological changes seen in AD. The expression of AHI1 promotes the intracellular translocation of APP and inhibits the amyloidosis process of APP, thereby decreasing the level of cellular Aβ in an *in vitro* model of AD (Ting et al., 2019).

Recent research has demonstrated that the movement of endosomes and vesicles carrying APP and Trks is blocked in mouse models of AD (Lazarov et al., 2007). APP interacts with Trks to modulate neurotrophic signaling and to facilitate neuronal survival and differentiation (Zhang et al., 2013). HAP1 plays a crucial role in stabilizing and transporting Trks. Specifically, HAP1 helps maintain appropriate levels of membrane TrkA by inhibiting the degradation of internalized TrkA (Rong et al., 2006). In addition, HAP1 deficiency leads to reduced TrkB levels and reduced survival of hypothalamic neurons in postnatal mice (Xiang et al., 2014). HAP1 and AHI1 interact with Trks, and knockdown of AHI1 or HAP1 results in decreased levels of TrkB, p-TrkB, and p-Erk in neurons and neurodevelopmental defects in mice (Ting et al., 2019). In conclusion, an in-depth study of the effect of HAP1 on APP intracellular transport is necessary to elucidate their role in the disease-mechanism of AD. Modulating APP-related transport mechanisms might be a promising technique for the treatment of AD, with HAP1 potentially playing a prominent role.

#### 4.1.3 Other neurodegenerative diseases

In addition to HD and AD, HAP1 has been highly associated with other neurodegenerative diseases. Abnormal amplification of the polyQ repeat sequence of TBP leads to TBP overaccumulation and the formation of intranuclear aggregates, which results in hereditary spinocerebellar ataxia 17 (SCA17) (Nakamura et al., 2001). In the context of aberrant TBP expression, the HAP1-TBP interaction may play an important role in counteracting TBP-mediated cytotoxicity. In patients with SCA17, TBP accumulates in the nucleus, leading to neuronal apoptosis (van Roon-Mom et al., 2005). In contrast, TBP does not readily aggregate in the nuclei of neurons expressing HAP1/STB, which may protect neurons against SCA17-related pathological changes (Prigge and Schmidt, 2007). HAP1 prevents the formation of nuclear aggregates and exerts neuroprotective effects in the presence of aberrant TBP accumulation. In addition, HAP1/STB can interact with and alter the physiological function of normal ataxin-3. HAP1 is also involved in pathological alterations of SCA3 through mutant ataxin-3 (Takeshita et al., 2011). Spinal bulbar muscular atrophy (SBMA) is closely associated with aberrant amplification of the repetitive sequence of CAG in exon 1 of the androgen receptor (AR) gene (Arnold and Merry, 2019). HAP1 interacts with/ and forms inclusion bodies with AR via its ligand-binding structural domains in a way that depends on the length of the polyQ tract (Takeshita et al., 2006). One study reported that ARQ65-induced apoptosis is inhibited by co-transfection with HAP1. This finding suggests that HAP1 acts as an important intrinsic neuroprotectant to reduce apoptosis and is closely related to the pathogenesis of SBMA.

### 4.2 Neurological diseases associated with HAP1

#### 4.2.1 Pain

Neuropathic pain is a type of intractable pain caused by injury to the sensory nervous system, the pathogenesis of which is related to peripheral hyperexcitability and central sensitization (Finnerup et al., 2021). Existing studies have shown that many HD patients exhibit large afferent delays and abnormal spinal pain responses (Mirallave et al., 2017; Li et al., 2023). The same phenomenon can also be observed in mouse models of HD, as these animals exhibit diminished pain responses in response to inflammatory stimuli (Lin et al., 2018). As a protein that has an important relationship with HD, HAP1 is highly involved not only in HD but also in pain progression.

According to the previous section, HAP1 is abundantly expressed in the spinal cord dorsal horn and DRG, which are considered the “major sensory centers” (Islam et al., 2017, 2020). HAP1 expression is also significantly increased in the dorsal horn and DRG in mouse models of neuropathic pain (Pan et al., 2023). This suggests that HAP1 may have a substantial impact on the regulation of nociceptive/ proprioceptive functions, which indicates that its pathophysiological role in pain and even itching might be important. HAP1 is associated with the transport and endocytosis of pro-BDNF and BDNF (Wu et al., 2010). Previous studies have shown that BDNF can act as a modulator of nociceptive perception (Hopp, 2021). BDNF is produced in the DRG and transported to the dorsal horn (Yang et al., 2011), where it is involved in pain sensitization and transduction (Cao et al., 2020; Zhou et al., 2021). Knockdown of HAP1 reduces the surface expression of Cav1.2 channels in sensory neurons, which in turn reduces Ca^2+^ influx into sensory neurons after nerve injury and ultimately reduces BDNF expression and secretion (Pan et al., 2023). HAP1 deficiency inhibits neuronal excitability and reduces pain. In a sciatic nerve injury model, it was shown that HAP1 knockdown may regulate the activation of astrocytes and microglia, which would inhibit the inflammatory response after nerve injury and ultimately curtail the progression of neuropathic pain (Pan et al., 2023). A genome-wide association study of fibromyalgia syndrome revealed a significant association between pain and the HAP1 gene, with HAP1-deficient mice exhibiting mechanical allodynia and nociceptive hypersensitivity suppression in both acute and chronic pain models (Gloor et al., 2022). In addition, other evidence suggests that HAP1 participates in the regulation and transmission of nociceptive sensations. HAP1 regulates the recruitment of TrkA receptors to maintain the survival of sensory neurons and promote neurite development (Rong et al., 2006). HAP1 is also involved in the recruitment of GABA_A_Rs, which contributes to neuronal excitability and the transfer of pain signals (Kittler et al., 2004). Several studies have shown that the number of GABAergic neurons is reduced in individuals with neuropathic pain, while elevated levels of GABA and GABA_A_Rs may reverse and reduce pain (St John and Smith, 2018; Zeilhofer et al., 2018). In conclusion, in-depth studies on HAP1 and pain as well as determining the role of HAP1 and its underlying mechanisms in pain development will help to identify both new strategies for managing neuropathic pain and new therapeutic targets.

#### 4.2.2 Spinal cord injury

Spinal cord injury (SCI) is defined as neurological dysfunction or permanent loss of spinal cord functions (including sensory, motor, and autonomic functions) below the plane of injury caused by external direct or indirect factors that manifests as varying degrees of paralysis (Eli et al., 2021). The pathological process of SCI is intricate and involves multiple factors, such as neuronal death, edema, axonal injury, demyelination, inflammation, and microcirculatory disorders (Okada, 2016).

Huntingtin-associated protein 1 is specifically expressed in spinal cord neurons, as more than 90% of neurons in layers I–II and X as well as neurons in the autonomic preganglionic area express HAP1 (Islam et al., 2017), which functions in neuronal development and differentiation. HAP1 is also involved in various receptor-regulated cycles and transports to promote neuronal survival and differentiation (Lim et al., 2018). HAP1 knockout mice exhibit substantial degenerative brain lesions, neuronal apoptosis, and developmental disorders (Dragatsis et al., 2004; Xiang et al., 2014). After SCI, HAP1 can promote neurological recovery and neurite growth during the differentiation of neural stem cells (NSCs) *in vitro* (Miao et al., 2023). The performance of mice in a model of SCI significantly improved after the intrathecal injection of recombinant HAP1, as evidenced by cortical locomotor evoked potential (LEP) scores, pain sensation, and temperature sensation (Miao et al., 2023). Similarly, studies in our laboratory revealed that the expression of HAP1 decreased dramatically after SCI. HAP1(+/−) mice showed a significant reduction in autonomic recovery from SCI and neurite protrusion growth was significantly inhibited when HAP1 was silenced in VSC4.1 cells(anterior pedunculated motor neurons of the spinal cord). Activation of the MAPK-ERK1/2 pathway in NSCs promotes the process of neurite outgrowth (Wang Y. et al., 2021), and signaling pathway array and Western blot analyses have revealed that recombinant HAP1 strongly induced TrkA-MAPK/ERK phosphorylation to promote neurite outgrowth and neurological recovery in NSCs (Miao et al., 2023). In conclusion, HAP1 promotes neuronal differentiation at the site of SCI and the growth of NSCs during axonal regeneration through the TrkA-MAPK pathway, and therefore, this strategy might improve the prognosis of SCI in mice. Additional research on the impact of HAP1 on SCI may enhance our understanding of the mechanisms by which HAP1 may be used in the treatment of SCI and provide novel treatment strategies.

#### 4.2.3 Epilepsy

An imbalance between neuronal excitation and inhibition due to failures in neurotransmission is widely recognized as an etiological factor in epilepsy (Li et al., 2022). GABA_A_Rs are considered among the most important receptors involved in the induction of epilepsy. Disruption of synaptic aggregation and a reduction in the number of GABA_A_Rs at the membrane lead to excitatory potentiation and abnormal network oscillations in the neuronal circuits of epileptic patients (Tipton and Russek, 2022). As a key mediator in the transport of GABA_A_Rs, HAP1 interacts directly with GABA_A_Rs, prevents lysosomal degradation of internalized GABA_A_Rs and facilitates the recirculation of internalized GABA_A_Rs to synapses, which is critical for controlling rapid inhibitory synaptic transmission. Reduced HAP1 expression attenuates the transport and synaptic inhibition of GABA_A_Rs (Kittler et al., 2004).

Huntingtin-associated protein 1 was shown to regulate seizure by controlling the activity of GABA_A_R in animals with pentylenetetrazole (PTZ)-induced epilepsy (Li et al., 2020). In the brains of epileptic rats, the expression of GABA_A_Rβ^2/3^ and HAP1 is reduced, and the HAP1-GABA_A_Rβ^2/3^ complex is disrupted. In contrast, the upregulation of HAP1 in PTZ-induced seizures could increase the expression of surface GABA_A_Rβ^2/3^ and the amplitude of miniature inhibitory postsynaptic currents (mIPSCs), which exert antiepileptic effects (Li et al., 2020). After further exploration of its detailed network of molecular interactions, HAP1 was shown to specifically interact with the chaperone protein 14-3-3 to form a cargo adapter complex that regulates the expression of surface GABA_A_Rs and the amplitude of mIPSCs (Wen et al., 2022). In epilepsy, disruption of the HAP1/14-3-3 complex reduces the strength of GABA_A_R-mediated inhibition of synaptic transmission (Wen et al., 2022). Elucidating the critical role of HAP1 in influencing inhibitory synaptic responses in epilepsy is relatively important, as HAP1 may serve as a promising therapeutic target for epilepsy.

#### 4.2.4 Ischemia

One of the pathological features of cerebral ischemia is an imbalance in excitatory/inhibitory signaling and neuronal death (Ghozy et al., 2022). Disruption of GABA_A_R synaptic aggregation and a reduction in GABA_A_R surface expression are believed to contribute to the altered balance between excitatory and inhibitory neurotransmission in the ischemic brain (Fritschy and Panzanelli, 2014). Downregulation of GABA_A_R recycling can lead to neuronal death in cerebral ischemia *in vitro* (Costa et al., 2016). HAP1 specifically interacts with the GABA_A_R subunit and regulates the level of GABA_A_R at synapses (Kittler et al., 2004).

Oxygen/glucose deprivation (OGD) can be used to construct a whole-brain ischemia model, and in this model, the recirculation of the GABA_A_R β3 subunit, the interaction between GABA_A_R and HAP1, and the total protein level of HAP1 are decreased. However, when hippocampal neurons are transfected with HAP1A or HAP1B, the OGD-induced decrease in the number of surface GABA_A_R β3 subunits is abrogated, and the survival of hippocampal cells is improved (Mele et al., 2017). In that study, HAP1A demonstrated greater therapeutic potential than HAP1B in protecting hippocampal neurons in the animals subjected to OGD (Mele et al., 2017), which may be related to the enrichment of HAP1A at the tips of neuronal synapses. Considering the protective role of GABA_A_R stabilization against ischemia-induced neuronal death (Smith et al., 2012), increasing GABA_A_R recirculation via HAP1 is crucial for the treatment of cerebral ischemia.

Another study revealed that HAP1 is highly important for the protective effect of dexamethasone in ischemic brain injury. As a class of clinical glucocorticosteroids, dexamethasone reduces infarct volume and protects neurological function in ischemic stroke patients (Bertorelli et al., 1998; Knox-Concepcion et al., 2019). In one study, the level of HAP1 in the brains of mice significantly increased after dexamethasone treatment (Xin et al., 2021). Dexamethasone can regulate HAP1 expression by affecting GR levels, and this finding is consistent with previous studies reporting that have reported that HAP1 colocalizes and interacts with the GR in mouse hypothalamic neurons (Chen et al., 2020). Further studies showed that HAP1 modulates the levels of pTrkB, pAkt, and pErk 1/2 after ischemic damage and dexamethasone therapy. Dexamethasone shields against ischemic brain damage by increasing HAP1 to inhibit the pAkt signaling pathway and promote the pErk signaling pathway (Xin et al., 2021). HAP1 is proposed as a promising therapeutic target for the treatment of cerebral ischemia.

#### 4.2.5 Depression

Depression is the predominant neuropsychiatric symptom in patients with HD and affects 30%–70% of individuals (Bilal et al., 2022). Clinical depression is one of the most prevalent mental disorders worldwide, and its etiology is not completely clear (Elias et al., 2022). Various hypotheses have been proposed, including reduced 5-HT levels, synaptic dysfunction, hyperactivity of the hypothalamic-pituitary-adrenal axis, and altered expression of BDNF (Peng et al., 2015). Recently, hippocampal neurogenesis has been found to be closely related to the pathogenesis of depression (Eisch and Petrik, 2012; Hill et al., 2015; Tartt et al., 2022). As mentioned earlier, HAP1 mediates the intracellular transport of various neurotrophic factors and their receptors and plays a crucial role in neuronal survival and differentiation. HAP1 stabilizes the level of the stem cell factor receptor c-kit, and the deletion of HAP1 leads to the downregulation of c-kit and impaired neurogenesis in the hippocampal dentate gyrus during the postnatal period, which results in an increased predisposition to depressive-like behaviors in both adolescent and adult mice (Xiang et al., 2015). Increased expression of c-kit in the hippocampus of mice after birth promotes hippocampal neurogenesis and ameliorates symptoms of depression. Moreover, HAP1 is involved in hypothalamic neurogenesis via regulation of the BDNF/TrkB pathway and other mechanisms. Depressive symptoms caused by AHI1 loss can be alleviated by overexpression of TrkB in the amygdala (Xu et al., 2010). HAP1 controls postnatal hippocampal neurogenesis and adult depressive behaviors via a novel mechanism, which suggests potential new approaches for depression prevention and therapy.

Impaired GR signaling is a significant factor in stress-related diseases, particularly depressive disorders (Anacker et al., 2011a; Dean and Keshavan, 2017), and targeting GR receptors as a treatment has been shown to effectively regulate hippocampal neurogenesis and alleviate depressive symptoms (Anacker et al., 2011b,^2013^; Thiagarajah et al., 2014). GR levels are also regulated by HAP1, which interacts with and stabilizes GR in the cytoplasm of mouse hypothalamic neurons. Depletion of HAP1 reduces the GR expression level in the hypothalamus and shortens the half-life (Chen et al., 2020). In addition, AHI1, which belongs to a class of proteins that interact with HAP1 for mutual stabilization (Sheng et al., 2008), interacts with GRs so as to stabilize each other in the cytoplasm (Wang B. et al., 2021; Wei et al., 2024). Significantly, AHI1 deficiency promotes the degradation of GRs in the cytoplasm and reduces the nuclear translocation of GRs in response to stress (Wang B. et al., 2021). The absence of AHI1 in neurons leads to a depressive-like phenotype and decreased sensitivity to antidepressants (Xu et al., 2010; Wang B. et al., 2021). Increased levels of the mitochondrial AHI1/GR complex improve depressive behaviors (Wang et al., 2023). These findings have elucidated the relationship between HAP1 dysfunction and stress, and provide strong support for the development of new therapies for HAP1-related diseases.

#### 4.2.6 Tuberous sclerosis complex

Tuberous sclerosis complex (TSC) is an autosomal dominant multisystemic genetic disorder. The neurological manifestations of TSC include epilepsy, autism, and cutaneous damage (Randle, 2017; Portocarrero et al., 2018). In TSC, mutations in the TSC1 or TSC2 gene result in hyperactivation of mechanistic target of rapamycin (mTOR), which ultimately causes neuronal degeneration. mTOR plays an important role in the regulation of cellular functions by forming two unique multiprotein complexes, mTORC1 and mTORC2 (Uysal and Şahin, 2020). The pathogenesis of autism and mental retardation is associated with dysregulation of the mTORC1 pathway (Santini et al., 2013). Proteomic methods have revealed that HAP1 is a novel functional associate of TSC1 (Mejia et al., 2013), and reducing HAP1 expression in hippocampal neurons leads to decreased TSC1 levels and triggers activation of the mTORC1 signaling pathway. Inhibition of mTORC1 activity attenuates the hippocampal neuronal phenotype induced by HAP1 knockdown (Mejia et al., 2013). HAP1 controls the location and characteristics of axons in hippocampal pyramidal neurons, which enhances the connection between axon-dendrite polarization and neuron placement. The novel function of HAP1 in regulating neuronal mTORC1 signaling has important implications for understanding cognitive developmental disorders.

#### 4.2.7 Down syndrome

Trisomy 21 occurs when an additional chromosome 21 is present; this leads to a set of clinical characteristics known as Down syndrome (DS), which is characterized by growth and developmental delays, abnormal facial features, and cognitive deficits (Antonarakis et al., 2020). No efficacious treatment is currently available. Dual-specificity tyrosine phosphorylation-regulated kinase 1 A (DYRK1A), located on chromosome 21, is an important candidate gene associated with DS and is expressed at relatively high levels in individuals with DS (Stagni and Bartesaghi, 2022). Transgenic mice with elevated levels of DYRK1A display cognitive and behavioral alterations resembling those observed in individuals with DS (Altafaj et al., 2001; Moyer et al., 2021).

Mass spectrometry has revealed that HAP1 interacts with DDB1 and CUL4-associated factor 7/WD40 repeat 68 (Dcaf7/WDR68) in STBs, which regulates Dcaf7/WDR68 levels and nuclear translocation (Xiang et al., 2017). Dcaf7 is required for normal DYRK1A expression (Yousefelahiyeh et al., 2018). Furthermore, HAP1 competes with DYRK1A in the cytoplasm for binding to Dcaf7, while depletion of HAP1 promotes DYRK1A-Dcaf7 interactions and increases DYRK1A protein levels, which ultimately leads to neurodevelopmental delays and weight loss in postnatal mice (Xiang et al., 2017). Previous studies have also reported that HAP1(−/−) mice with severe degeneration of hypothalamic neurons exhibit feeding deficits and weight loss and die soon after birth (Chan et al., 2002; Li et al., 2003; Xiang et al., 2014). Surviving mutants also exhibit slow growth (Dragatsis et al., 2004). DYRK1A has been proposed to control the interaction between HAP1 and Dcaf7, which influences postnatal development. By focusing on HAP1 or Dcaf7, it is possible to improve growth delays in DS. In addition to growth retardation, cognitive developmental deficits are also important clinical symptoms in children with DS (Zhang et al., 2014). Although no definitive study has been published on the association of HAP1 with cognitive impairment in individuals with DS, existing studies suggest that the two are inextricably linked. Under normal conditions, AHI1 and GR stabilize each other (Wang B. et al., 2021), inhibit GR nuclear translocation, promote the binding of AHI1 to Dcaf7, and inhibit the binding of DYRK1A to Dcaf7. In contrast, AHI1 deficiency promotes the binding of DYRK1A to Dcaf7, which leads to elevated DYRK1A, reduced synaptic plasticity, and cognitive deficits (Wei et al., 2024). HAP1 interacts with and stabilizes both GR and AHI1. HAP1 deficiency not only results in decreased GR expression in mouse hypothalamic neurons but also leads to a significant reduction in AHI1 levels (Sheng et al., 2008; Chen et al., 2020). HAP1 is expected to impact the development of cognitive impairment in individuals with DS through the GR and AHI1. Collectively, these studies might provide novel insights for targeting HAP1 or Dcaf7 as a potential therapy for DS.

### 4.3 Type 2 diabetes mellitus

The prevalence of diabetes is greater in the HD patient population (Podolsky et al., 1972; Podolsky and Leopold, 1977). Pancreatic β-cells in mouse models of HD using R6/2 mice also develop functional defects that ultimately lead to diabetes (Björkqvist et al., 2005; Montojo et al., 2017). Abnormal interactions between mHtt and β-tubulin disrupt vesicular transport and insulin secretion (Smith et al., 2009). HAP1, a class of proteins tightly associated with Htt, may also contribute to the disruption of insulin vesicle transport and insulin secretion in HD-associated diabetes.

Abnormal insulin secretion is necessary for the development of type 2 diabetes mellitus (T2DM). HAP1 is selectively expressed in rat pancreatic β-cells (Liao et al., 2010), while knockdown of HAP1 in mouse pancreatic β-cells results in lower plasma basal insulin levels in KO mice than in controls (Pan et al., 2016), impaired glucose tolerance and reduced insulin release (Cape et al., 2012). HAP1A serves as an adapter that connects actin-based myosin Va motor protein with insulin-containing vesicles, which controls the transport of insulin-containing granules in pancreatic β cells (Wang et al., 2015). Reduced HAP1 expression hinders the binding between myosin Va and insulin-containing vesicles, impairs vesicle recruitment to the cell surface, and ultimately inhibits glucose-mediated insulin secretion (Cape et al., 2012). In addition, Ca^2+^ influx mediated by L-type Ca^2+^ channels directly stimulate the transport of secretory particles and triggers insulin exocytosis, which is crucial for insulin secretion (Correa et al., 2023). HAP1 suppression slows Cav1.2 intracellular trafficking, downregulates Cav1.2 surface expression, reduces Ca^2+^ influx, and ultimately affects insulin secretion by islet beta cells (Pan et al., 2016). A series of studies on the role of HAP1 in insulin release may provide a new therapeutic target for metabolic disorders that are predominantly characterized by defective insulin release.

Another study on the role of HAP1 in adipocyte glucose transporter isoform 4 (GLUT4) translocation provided a new perspective on the relationship between HAP1 and T2DM. As the major insulin-sensitive glucose transporter, GLUT4 plays a critical role in controlling glucose homeostasis in vivo (Fischer et al., 2019). Impaired GLUT4 transport can lead to insulin resistance and T2DM (Czech, 2017). HAP1 forms a protein complex with GLUT4 and sortilin and participates in the insulin-stimulated translocation of GLUT4 in adipocytes (Gong et al., 2020). Reduced HAP1 expression disrupts GLUT4 transport, leading to impaired glucose homeostasis, subsequent insulin resistance, and ultimately, the development of T2DM (Gong et al., 2020). Elucidation of this mechanism could provide new insights into the pathogenesis of insulin resistance.

In addition, an as yet uncharacterized association may exist between HAP1 and T2DM. As an important antidiabetic factor, GABA has been found to regulate glucose-stimulated insulin secretion in a concentration-dependent manner in pancreatic β-cells (Indrowati et al., 2017; Hosseini Dastgerdi et al., 2021). Decreased HAP1 expression in the hypothalamus affects feeding behavior by downregulating the GABAR (Sheng et al., 2006). As BDNF is known as a “metabolic factor,” BDNF impairments are closely associated with increased appetite, obesity, and T2DM (Rozanska et al., 2020). BDNF is also decreased in the blood of T2DM patients (Bazyar et al., 2023). BDNF supplementation or enhancement of its downstream signaling pathways is an effective treatment for T2DM (Chan et al., 2019). Phosphorylation of synaptophysin I is also involved in inducing insulin-controlled cytokinesis in pancreatic β-cells (Yamamoto et al., 2003). HAP1 is crucial for both the recycling of GABA receptors and the transport of BDNF and synaptophysin. In conclusion, further exploration to decipher the association between HAP1 and T2DM will provide new therapeutic directions for diabetes mellitus and other related endocrine diseases.

### 4.4 Cancer

Cancer is rarely reported on the death certificates of individuals with HD (Sørensen et al., 1999; Bragina et al., 2023). In those with HD, the length of the mHtt repeat sequence is negatively correlated with cancer incidence (Murmann et al., 2018a,b). Recently, the relationship between HAP1 and certain types of cancer has been increasingly recognized and highlighted, warranting further research and discussion.

The expression of HAP1 is much lower in tumor tissues of breast, gastric, and pancreatic cancers than in normal tissues and benign proliferative tissues such as polyps (Zhu et al., 2013; Li et al., 2019; Qu et al., 2023). For example, in the pancreas, HAP1 is consistently expressed in pancreatitis and normal pancreatic tissues but is significantly decreased or undetectable in pancreatic cancer tissues (Li et al., 2019). These findings suggest that HAP1 can serve as a diagnostic biomarker for malignant tumors, including breast cancer and pancreatic cancer.

In addition to its role as a cancer biomarker, HAP1 is most likely a tumor suppressor. HAP1 promotes apoptosis and controls the malignant transformation of tumor cells. In one study, HAP1 overexpression in both breast cancer cell lines (MCF-7 and MDA-MB-231) and gastric cancer cell lines (MKN28 and AGS) significantly inhibited tumor cell growth and proliferation and reduced invasion and migration (Zhu et al., 2013; Qu et al., 2023). A similar study revealed that the HAP1 gene could also regulate the radiosensitivity of the breast cancer cell line MCF-7 and promote the apoptosis of MCF-7 cells after radiotherapy (Wu et al., 2015). To explore the possible underlying mechanism, we analyzed apoptosis-related pathways and found that the expression of apoptosis-related proteins, such as Bcl-2, Bax, caspase-3/9, and survivin, was significantly altered after HAP1 transfection (Zhu et al., 2013; Wu et al., 2015; Qu et al., 2023). These proteins are located downstream of the apoptosis signaling cascade and have crucial functions in apoptosis. The activation of Insp3R by HAP1 leads to an increase in Ca^2+^ release, which then triggers the activation of calpains. This subsequently induces the activation of caspase-3 and a cascade of proapoptotic proteins, which ultimately results in apoptosis (Zhao et al., 2022). Notably, the absence of ERs in MDA-MB-231 cells dose not induce HAP1 assembly, which results in an insignificant proapoptotic impact on MDA-MB-231 cells (Zhu et al., 2013). These findings suggest that HAP1 is involved in and enhances apoptosis by influencing the expression of apoptosis-related proteins.

Furthermore, as a desirable outcome of targeted glucose metabolism for anticancer therapies (Cui et al., 2023), glucose deprivation-induced cell death is more pronounced in gastric cancer cells that overexpress HAP1 (Qu et al., 2023). In HAP1-overexpressing gastric cancer cells, glucose deprivation leads to a significant decrease in ATP production and a significant increase in ROS production and triggers oxidative stress, which ultimately results in cell death (Qu et al., 2023). Currently, the way in which HAP1 targeting leads to tumor suppression is not well understood. Nevertheless, the combination of HAP1, caloric restriction, and ROS inducers may be a promising treatment strategy.

## 5 Conclusion and prospects

In summary, HAP1 interacts with a variety of proteins to regulate vesicular transport, membrane receptor translocation, gene expression, and cytoskeletal remodeling. HAP1 is also a potential therapeutic target for various neurological diseases. Studying the function of HAP1 and the proteins with which it interacts, as well as gaining a deeper understanding of the important role of HAP1 in various diseases, can provide new insights and research ideas for the treatment and study of neurological disorders, cancer, diabetes, and other related diseases.

## Author contributions

YJW: Writing – original draft, Writing – review & editing. YFW: Writing – original draft. YL: Writing – original draft. JY: Writing – original draft. HZ: Writing – original draft. RY: Writing – original draft. JP: Writing – original draft, Writing – review & editing.
